# Community-Based Participatory Research: Partnering with College Students to Develop a Tailored, Wellness-Focused Intervention for University Campuses

**DOI:** 10.3390/ijerph192316331

**Published:** 2022-12-06

**Authors:** Makenzie L. Barr, Jade McNamara

**Affiliations:** 1 Department of Dietetics and Human Nutrition, College of Agriculture, Food and Environment, University of Kentucky, Lexington, KY 40506, USA; 2 Department of Food Science and Nutrition, University of Maine, Orono, ME 04473, USA

**Keywords:** program development, community-based, college students, health-related quality of life

## Abstract

College students face unique challenges with leading healthful lifestyles. Using a community-based participatory research approach, college student research partners at two land-grant universities collected data and developed a tailored intervention to improve the well-being of college students. To inform the design of the program, college students were trained to conduct a needs assessment that included a campus-wide survey on the health behaviors of college students, environmental audits of health policies and food pantries on campus, and stakeholder interviews with campus health professionals. Outcomes of the needs assessment data highlighted university students ranked their health as “good” but nutrition health as “fair/poor.” Low or very low food security was self-reported by 36.9% of participants and had an overall diet quality score of 47.6 ± 10.1 out of 100. Health professional interview data indicated campuses provide healthful resources to students, but students are not aware those resources exist. Utilizing the needs assessment data previously mentioned, the nominal group technique was then used for student research partners to collaboratively determine the best intervention approaches and develop a wellness program. Student partners identified (1) education, (2) sharing of campus resources, and (3) incentives as important areas of intervention. Using the data collected, the student research partners developed a program titled, The College Cooking Connection, to address health-related quality of life in college students. Using a community-based participatory research approach to program planning, educators and researchers have a greater likelihood of addressing the current needs of the population they are targeting and developing a successful intervention to meet those health concerns. This study aims to partner with young adult university students to understand the college environment and allow the target community to be involved with the development of intervention programs for their campus.

## 1. Introduction

The transition to young adulthood has been additionally compounded by increasing rates of childhood obesity [1] and the incidence of mental illness in children and adolescents [2]. Improving the overall health of the upcoming generation is imperative to prevent chronic disease in future adults and to help ensure an able and active workforce and economic stability. Approaches to attenuating poor lifestyle behaviors of young adults can be advanced through working with a captive audience of over 12 million young adults (aged 25 or younger) college students that are attending university in the United States [3]. Unfortunately, the transition to college is typically associated with unfamiliar independence and, thus, an onset of individual behavior choices that can impact health. As young adults progress through college, many participate in unhealthy behaviors that lead to unintended consequences. On average, young adults gain approximately 3–4.3 kg in their first year at university [4,5]. This weight gain puts young adults at a higher risk of obesity and related co-morbid conditions by age 35–37. Encouragement and facilitation of beneficial behaviors such as healthy eating, physical activity, and good mental health practices through lifestyle behavior changes assist young adults in risk reduction of developing chronic diseases later in life [4].

With this unfamiliar transition through independence and responsibility, levels of stress and, consequently, poorer mental health may be additional unintended consequences [6]. In 2017, the prevalence of any mental health illness (AMI) was found to be the highest in young adults (25.8%) when compared to younger and older age groups [6]. Impaired mental health not only contributes to poor quality of life but also impacts overall dietary intake and physical abilities resulting in a greater risk of chronic disease [6]. A major contributor to poor mental health status among college students is stress and dealing with fatigue and anxiety due to the stressors of university academic and social life [7]. While a vast majority of students report having access to services to help with their physical and mental health, these negative effects on their mental health status are still prevalent [8]. When examining correlations with mental health symptoms, poor dietary quality has been linked. Within a young adult college population, positive correlations have been shown with the relationship of mental health symptoms with low fruit and vegetable intake, added sugar intake, and food insecurity status [8,9,10]. With young adults at greater risk of poor health outcomes [11], intervention strategies to improve the health-related quality of life of young adults are warranted [12], though increasing participation can be a challenge.

A way to improve the feasibility, sustainability, and buy-in of behavior change interventions is to involve stakeholders in the process and progress of designing, implementing, and evaluating programs. Community-based participatory research (CBPR) applies core principles that, as explained by Simona and colleagues, “(a) foster joint ownership in the identification of health priorities, the development, and evaluation of research strategies and their design, and the dissemination of findings; (b) a keen recognition and appreciation for the importance of stakeholder-driven priorities, research, and solutions; (c) building capacity of both stakeholders and researchers to engage in research collaboratively; and (d) recognizing that conducting the research is not the endpoint but continues on with a commitment to dissemination, spread, adoption, and sustainability” [13,14]. Community-based participatory research approaches have been shown to lead to sustainable health behavior change [15]. Among college student populations, the reduction of risky health behaviors has been a foci of many CBPR interventions, such as reducing excessive alcohol consumption, improving nutrition and dietary quality, reducing sedentary lifestyle, suicide prevention, reduction and awareness of substance abuse, among others [16,17,18]. Using a community-wide approach to health programming has also proven to have a positive long-term return on investment by reducing work absenteeism and healthcare costs associated with obesity and chronic disease [19]. These studies highlight that CBPR is both a feasible and successful approach when working with student partners to develop health programming for young adults [20].

Undergraduate students report wanting to make changes on their campus and wanting to be involved in bringing healthful changes to their communities [21]. Often students are at a loss for where to start or how to bring about change. Offering course-based research experiences, such as a CBPR hands-on course, gives students a chance to make their voices heard in health programming initiatives and will lead to greater support and student participation once programs come to fruition [22,23]. Based on these findings, there is evidence to support using a CBPR approach to develop culturally relevant and tailored health programs. The model of utilizing a college course for these efforts includes stronger buy-in of students to take ownership of the content and data. A study by Barr et al. utilized a similar approach to lead a college course for students who were interested in aiding with the development of a social marketing and environmental intervention [24]. Authors found that participating in the courses resulted in greater physical activity and less stress compared to a control group of students, and the social marketing campaign that was developed was tailored to their peers’ health needs [17].

Therefore, the current research aimed to utilize a CBPR training college course and college student research partnership to collect needs assessment data on the target population and serve as a community advisory board (CAB) to assist in the development of a peer-designed health and well-being intervention. From this collaboration, an intervention will be informed by collected needs assessment data, student interests and passion, and the overarching theme of sustaining health and promoting self-sufficiency post-graduation. Findings from the students’ needs assessment and interviews will be shared.

## 2. Materials and Methods

**Figure 1:**
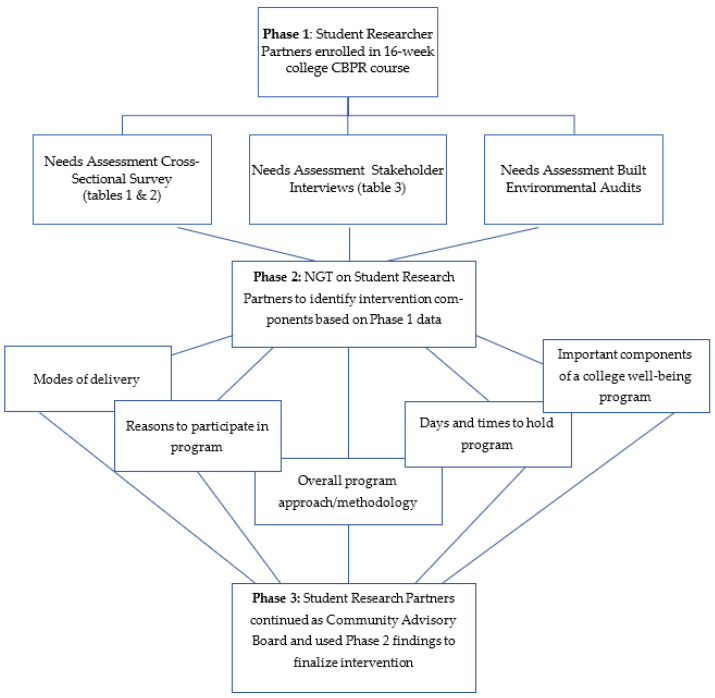
Flow diagram of study phases and data collection.

This research is broken into three phases (Figure 1). Phase 1 will focus on engaging student research partners through a CBPR course and a needs assessment. Phase 2 will focus on the process of identifying key programming methods to meet the health needs identified during Phase 1. Phase 3 includes meeting a community advisory board (CAB) to finalize programming details. All methods were approved by the Intuitional Review Board at each of the participating universities (University of Maine (protocol code 2020-07) and the University of Kentucky (#61400)).

### 2.1. Phase 1: Community-Based Participatory Research Course for Undergraduate Student Research Partners

At each participating university, undergraduate students were recruited across campus to participate in a special topics course in the fall of 2021, also referred to as Phase 1. Inclusion criteria for the course included being a student at the university for at least 1 year and between the ages of 18 and 24. The 3-credit hour course, entitled “Community-Based Participatory Research: An Experiential Learning Course to Improve Your Campus Environment” (CBPR Course), was co-taught by principal investigators trained in young adult community health, CBPR methodology, qualitative and quantitative data collection and management, and program implementation. The course was modeled from an existing CBPR course shown to be successful in engaging undergraduate students in community-based programming [25]. Throughout the 16-week semester course, students were trained with hands-on experience on the principles of CBPR, including behavior change theories, conducting ethical research, mixed methods data methodologies and analyses, development of data collection measures, conducting a needs assessment, conducting interviews with key stakeholders, and learning to compile data into an easy-to-use format for scientific communication. For the hands-on experience of utilizing these skills, students were ultimately charged with serving as student research partners to assist in identifying health-related needs of their campus through collection of needs assessment data.

#### 2.1.1. Student Led Campus Health Needs Assessment

Formative data collection was designed utilizing CBPR principles to partner with student researchers to determine approaches to collect needs assessment data about the health of their university campus. The needs assessment data was collected by the student researchers enrolled in the CBPR course, including a cross-sectional campus health survey, stakeholder interviews, and an environmental audit.

#### 2.1.2. Campus-Wide Cross-Sectional Needs Assessment Survey

A cross-sectional survey was developed by the student research partners to survey their fellow students at each campus. Students who were between the ages of 18 to 24, able to read and understand English, and were currently enrolled as a student at the university, were recruited to take this formative cross-sectional survey. Recruitment materials were sent through email distribution to undergraduate student listservs. Data was housed on the principal investigator’s Qualtrics account and de-identified for the student researchers to use for summation. At each university, this cross-sectional campus-wide survey was sent out to capture formative information on self-reported measures from college students’ including self-reported anthropometrics (height and weight), perceived general health and perceived nutrition health, hours of sleep and sleep quality, diet quality, food security, perceived stress, and cooking confidence [26,27,28,29,30].

Self-reported height and weight were used to calculate body mass index and has been identified as a reliable self-reported measure among young adults [26]. One item from the CDC’s Healthy Days Module was included in rating their perceived general health on a Likert item scale (1 as excellent through 5 as fair) [30]. Sleep hours and sleep quality (i.e., “during the past month, how would you rate your overall sleep quality?”) were assessed using the validated Pittsburgh Sleep Quality Index, a 19-item tool validated in this population [31]. The short Healthy Eating Index is a 22-item survey to identify the dietary quality of students through an algorithm calculating the percentage that their diet aligns with the Dietary Guidelines for Americans (scale of 0–100% with a higher number indicating diet quality aligned with the guidelines) [27]. Perceived Stress Scale is a 10-item validated tool for understanding adults’ perceived stress through helplessness and lack of self-efficacy on a scale of 0–40 with categories indicating low (0–13), moderate (14–26), or high (27–40) stress [29]. The six-item food insecurity screener asks participants about their ability to obtain and consume appropriate and adequate amounts of food over the last 12 months [32]. Finally, cooking confidence questions were from the Food Preparation Knowledge and Confidence survey for young adults [33,34]. Students who completed the cross-sectional campus-wide survey were entered into a raffle to win a $50 Amazon gift card for their time. Data was housed on the principal investigator’s Qualtrics account and de-identified for the student researchers to use for summation.

#### 2.1.3. Campus Health Stakeholder Interviews

As part of the needs assessment data collection, each student research partner in the CBPR course identified and interviewed 2–3 health/wellness professionals on their campus that work directly with students or student health. Inclusion criteria required participants to be employed at their respective universities for at least 2 years and work with students in their daily jobs. Student researchers recorded and transcribed the interviews. Sample questions included, “Since you’ve been at the University, what areas of healthy living do you believe are well-done here for students, specifically” and “What do you think college students struggle the most with in terms of living a healthy lifestyle?”. Students in the course were trained in qualitative data analysis and worked to code the interviews and highlight common trends. During class meeting times, qualitative data trends were discussed, refined, and compared across universities to capture commonalities and differences.

#### 2.1.4. Environment Assessment

Lastly, student researchers decided to assess their built environment and chose to evaluate health policies that exist on campus [35] and the nutrition environment of the campus food pantries [36]. Food pantries were assessed through the Nutrition Environment Food Pantry Assessment Tool (NEFPAT). The NEFPAT scores are grouped by categories: 0–15 (Bronze), 16–31 (Silver), and 32–47 (Gold). Students also completed a policy audit from the POINTS tool that focuses on health-related policies that directly impact student health. The POINTS tool is summarized based on overarching health areas (nutrition, tobacco, alcohol, mental health). Students were asked to report whether there were full policies on campus, suggestions or recommendations of a behavior, or no evidence of a policy or suggestion.

Student partners utilized the collective data from the needs assessment, interviews, and built environmental assessments to develop their own health intervention independently, with the intention to share and collaborate on programming ideas during Phase 2. The needs assessment data included student health data (Healthy Eating Index, sleep, stress, nutrition literacy), interview data conducted with health and wellness professionals across their campuses, and environmental audit data (campus food pantry, healthful food availability, and health policies). Following the course, students were invited to attend a 3-day hybrid workshop to collaborate across universities to develop a shared pilot program to address health needs at each campus. 

### 2.2. Phase 2: Hybrid Program Planning Workshop

Undergraduate university students who participated in the CBPR course at each institution (Phase 1 students; n = 7) and invited university graduate students whose research area focuses on college student health and wellness (n = 3) participated in a 3-day hybrid workshop following the semester CBPR course. The workshop was used to bring student partners together from each participating university to share individually developed ideas for campus health interventions and collectively determine final plans for program implementation the following year.

At the hybrid workshop, Nominal Group Technique (NGT) was conducted with ten university students as participants. A series of questions were used to facilitate group discussion toward narrowing down the idea(s) for program organization and objectives [24]. Five questions were developed to narrow ideas for program components. NGT questions included: (1) techniques that would influence a college student to participate in a wellness program, (2) how often we should hold the events/how often would you attend events, (3) ranking top three topic areas/intervention ideas, (4) top two modes of intervention delivery for the program intervention, and (5) top idea that you believe your peers would participate in. By question, student partners were asked to take a minute to themselves to think about their answers. Each participant, round-robin style, was then asked to individually share their thoughts aloud without group discussion. Once each participant shared their thoughts and ideas, discussions were employed between participants and ideas. Following discussions, participants were then asked to narrow down all ideas to rank in the top three key points. 

### 2.3. Community Advisory Board

Additionally, following the CBPR course (Phase 1) and the Hybrid Workshop (Phase 2), student research partners who were members of the CBPR course and participants of the Workshop were invited to continue as Community Advisory Board (CAB) members. The purpose of the CAB was to plan out the details of the wellness intervention and work collaboratively with the research team throughout the months leading up to and through the intervention. These details included program name, programming methods, assessment instruments, duration of programming, and incentives.

### 2.4. Data Analysis

#### 2.4.1. Phase 1: Needs Assessment Data

Campus-wide survey data of college students at each university collected by the student researchers were used to show measures of central tendencies (age, body mass index, hours of sleep per night, short Healthy Eating Index, servings of fruit per day, and servings of vegetables per day) and frequencies (gender, race/ethnicity, sexual identity, years in college, dining plan, living on or off campus, perceived health status, perceived nutrition health status, overall sleep quality, cooking confidence, food security status, and perceived stress).

Qualitative interview data from university health and wellness stakeholders were collated by question from the trained student researcher. Interviews were initially coded by student research partners. Transcripts were double coded by a second student researcher. If a common consensus about coding could not be made by the student research team, an additional researcher resolved code discrepancies until agreed upon. Illustrative quotes were chosen by the student to represent the main themes.

#### 2.4.2. Phase 2: NGT Findings

Data from the NGT were collected in real-time as discussion was ongoing. Two trained qualitative researchers led the NGT with the student research partners as the participants. One PI led the discussion and implementation of the NGT, while the second PI took notes verbatim that automatically populated onto a screen projector for all participants to see. Discussions were had about each of the options to share positives and challenges to each. During discussions, notes were taken to allow members to see each other’s thoughts visually. Following discussions, each member ranked their top three choices of intervention activities and components, and the researcher tallied up the votes automatically on the screen. The top three choices of each question were noted and are found in the results section.

#### 2.4.3. Phase 3: Community Advisory Board

Students from Phase 1 and Phase 2 who agreed to continue as a CAB member were invited to participate in monthly meetings to finalize program implementation logistics. Plans for program events and methods are in progress of development for a Spring 2023 implementation. Subsequent articles will follow to share program outcomes.

## 3. Results

### 3.1. Phase 1 Needs Assessment Data 

*Survey Data.* The survey data collected (n = 987) as part of the student-driven needs assessment is reported in Table 1 and Table 2. University 2 accounted for 6% of the total sample, health behavior data of interest were examined by university, and there was no significant difference, so the data were then combined into one data set. The majority of students were white (90.4%) and female sex (62.6%). The average body mass index (BMI) was 24.7 kg/m2, indicating a “healthy” range BMI. Most students reported identifying as heterosexual (70.8%), followed by bisexual (14.6%). The sample considered their general health to be good (43.6%), but over 40% reported their nutrition health as fair/poor. A large portion of the students also reported low or very low food security (36.3%) and a poor overall diet quality score of 47.6 ± 10.1 out of 100. However, perceived cooking confidence was high, with average scores ranging from 3.8–4 out of 5. Lastly, perceived stress was reported to be moderate (55%) and high (42%), with only about 3% reporting low perceived stress.

*Interview Themes.* Ten health/wellness professionals (90% female) were interviewed across both universities. Interviewee professional positions included Student Wellness, Sports Medicine, Recreation Center, Food Pantry Manager, Registered Dietitian for the campus dining, College Dean of Instruction, and wellness-focused undergraduate course instructors. The trends that emerged and were consistent across the two campuses included (1) lack of awareness of health-related programs or resources on campus, (2) need for mental health resources is large and capacity to meet the needs is limited, and (3) students lack life skills and coping skills related to health outcomes.

Lack of Program or Resource Awareness. Throughout campus, most interviewees believed that there are several resources available to students for health-related needs. However, though they believe there are enough resources to assist and support the study body, stakeholders shared that they believe students are unaware of these resources. One interviewee shared, “I do think that we have opportunities for students, but they often feel so overwhelmed in the midst of it all that they don’t have the wherewithal to go out and search for something, so we need to figure out how to get the information to them where we are, pretty much force it on them so they then realize oh okay I can do this.” Several other interviewees shared similar sentiments, including the lack of promotion of these resources from the host organizations or the lack of student knowledge to seek out the resources. One participant shared, “From my view, student don’t know what’s out there, so maybe encouraging students or directing them to resources that are available on campus because it’s not necessarily the lack of resources, but the lack of knowledge.”

Mental Health Needs and Lack of Care Capacity. An important conversation thread throughout interviews with stakeholders seemed to involve mental health care needs for these young adult college students. It was shared that, “The mental health stress of balancing everything, getting burned out, relationships, and homework expectations…can take a mental toll”. When expanding on the need for mental health resources, the understanding of the clinic capacity to care for all students came to light. One interviewee stated, “I do feel like students’ emotional health has gotten worse and that we’ve struggled here on campus to meet that demand and keep up with that incredible need. And I know that there are individuals on campus that are dedicating their whole entire lives to that and they’re trying very hard, it just seems that the need itself is so much greater than we ever anticipated so I think that emotional well-being is a need that we struggle to meet on campus.” In line with shared thoughts on appointment wait times and lack of accessibility, mental health was a vital conversation throughout the interviews that is believed to need to be addressed.

Lack of Life Skills and Coping Skills. Shared by interviewees was the thought that students are lacking in areas of life management, including areas such as stress management, budgeting skills, cooking skills, and overall time management. As a holistic approach to overall well-being, one interviewee stated the influence of time and stress management sharing, “I to go back to the time management and stress management. People don’t know how to handle it, I think that that’s when people crack and you know, ultimately, you know your health is one of the things whether it’s for eating or you know I don’t feel like I have time to exercise and different things, so I think ultimately struck by going back to stresses, lead to a lot of unhealthy decisions so.” To build some of these life skills, one interviewee recommended, “I think that it would lean heavier on the resiliency in mental health. I think that a lot of students come into campus, and this is their very first time on their own, but they weren’t given the opportunity to learn certain life skills to help them manage stress, manage their time wisely, and also just build emotional resiliency”.

As an additional area of life skills, cooking capabilities and skills were highlighted. One interviewee shared that although they try to teach students cooking skills and preparation methods, they are not making the behavior change to cook for themselves, “So definitely I think teaching cooking skills, I think, is something that again we as a department are striving to find new ways to do that. So when students leave here like I mentioned before, we’re teaching them about the cuisines, they are trying new foods, they’re trying different ways of food preparation, but they’re not doing it themselves.” To overcome some of these issues, one interviewee suggested a life coach, sharing, “think every student, every person should meet with a health coach. Because it’s not a therapist or counselor, it’s just someone who can sit down with you and talk about all the areas of wellness and ask you what you’re wanting.” Another interviewee shared similar statements about being “proactive” in providing care and reducing the stigma around seeking resources before it would get to a detrimental point.

*Environmental Audit.* In addition to the cross-sectional campus survey and stakeholder interviews, the college student research partners additionally completed an environmental audit at each university individually. Items involved in the environmental assessment included campus health-related policies and the campus food pantry. In terms of health policies, it was found that both universities had wellness policies in place related to tobacco use and alcohol consumption. Though both universities did have numerous initiatives, pledges, and/or interventions on campus that focused on promoting healthful behaviors on campus, neither had written policies focused on health promotion, such as healthful eating, exercise, or mental health.

The NEFPAT assessment consisted of six objectives focused on different healthful eating environment opportunities. Each of the six objectives has different strategies, and one point is earned per strategy that is implemented. Scores are grouped by categories: 0–15 (Bronze), 16–31 (Silver), and 32–47 (Gold). Both universities scored within the ‘Silver’ category, with student research partners scoring their pantries on a range of 17–19 at university 1 and 18–19 at university 2.

### 3.2. Phase 2: NGT Outcomes

Thirteen individuals attended the 3-day workshop, 2 PIs, 3 graduate researcher assistants well versed in health/wellness programming, and 7 undergraduate student partner participants. Prior to the NGT, each student research partner that was enrolled in the Phase 1 CBPR course put together a program pitch for an intervention to run on their campus to improve college student health. Each student research partner presented their pitch at the workshop. From the information shared in the program pitches, overarching questions were used to determine a final consensus on the program topic and focus through the NGT. Ten university students participated in the NGT led by two trained qualitative researchers. Student participants were asked a series of four overarching questions regarding their top choices in program logistics (e.g., motivation for participating in programs, time and day of weeks they would attend, and important areas of intervention on their campus) (Table 3).

Participants indicated that the top three areas that would influence them and others to participate in on-campus events would include being incentivized (i.e., monetarily or with tangible items), the convenience of attending or engaging in program events (i.e., timing during the week, location), and understanding the personal benefit that they would receive from this program (i.e., building skills, learning something of value). Each student on campus has very different schedules, traveling capabilities, and personal responsibilities. When discussing the appropriate frequency of intervention components and timing throughout the week, participants indicated that they are more likely to attend events that are no more than two times per month and on weekday afternoons.

Regarding the top three areas of importance identified through their own experiences, data collection during the semester course, and synthesis of that data, participants identified the areas of (1) communication of campus resources, (2) nutrition knowledge and skills, and (3) mental health support. Participants agreed that there are many programs on campus that assist with various elements of health; however, there is a lack of communication of these events broadly across campus. Likewise, participants felt that students, overall, have a lack of knowledge of nutrition-related components of health and well-being and would benefit from enhanced knowledge and hands-on practicing nutrition-related skills (i.e., cooking demonstrations). To facilitate communication of these programs and resources, participants felt that modes of delivery to students included in-person events and online or texting communication and ranked a mixture of these modes as their top-ranking avenue for communication throughout the intervention.

Finally, as part of the workshop, each student partner presented their own idea for an intervention based on course materials, data collection, and data synthesis at their sites. From these presentations, participants in the NGT were asked to identify their top-ranked ideas for intervention events and components. In ranking order, participants chose (1) education, including nutrition, budgeting skills, grocery shopping knowledge, and cooking, (2) sharing of campus-related resources via text messaging, such as scheduling mental health appointments, food pantry hours and restocks, opportunities for physical activity, and other in-person events on campus, and (3) utilization of a “loyalty” punch card that would incentivize students to attend campus events and use student-related campus resources.

### 3.3. Phase 3: Community Advisory Board Outcomes and Program Plans

At the end of the workshop, all attendees (N = 13) were invited to continue with the development process of the wellness program. Ten out of 13 participants agreed to serve on a CAB to contribute to the design and details of the intervention. The CAB met bimonthly for 1.5 h over the course of 8 months. The results of the CAB meetings included titling the wellness program College Cooking Connection to be implemented as a next step that will be described in a future manuscript. The College Cooking Connection will have a two-pronged approach to address health-related quality of life in college students. The first approach will be to host a four-lesson cooking course that focuses on improving diet quality and food security among college students. The second approach is a text messaging campaign to disseminate campus-related wellness tips and resources to students in an accessible way. The final program will be run on the university campus for a semester period during the school year (approximately 16 weeks) and will be advertised widely throughout the university for all to participate.

Within the four-lesson cooking course, students will be taken through a curriculum focusing on healthful eating at college, budget cooking, and meal prepping. Program lessons will last approximately 1 h each and will be held in a centralized location on the university campus that includes a teaching kitchen set-up (stations of stoves, ovens, preparation spaces, sinks, etc.). Participants will be led by trained nutrition research personnel through educational materials and a cooking lesson.

The text message portion of the intervention will include educational awareness of resources on campus that are there to improve college student well-being. Each campus will partner with health-related organizations on campus to collate information and share upcoming events such as ways to schedule mental health appointments, where to access food pantries, and upcoming physical activity classes, among others.

## 4. Discussion

The current study aimed to (1) train students in the principles of CBPR to apply a student-driven approach to assess the healthfulness of their university campus environment and (2) utilize the NGT approach when partnering with students in the course to determine best practices for a peer-led, campus intervention. CBPR approaches to research endeavors are a way to enhance buy-in and trust from the community being examined, as well as improve the sustainability of implementation aspects. As college and young adulthood are a period of the lifespan with many points of influence and independence, seeking out novel ways to improve health and well-being is beneficial for improving involvement and retention of programming while also altering targeted health behaviors. Utilization of this method is being planned with the CAB student researchers, and they have plans for implementation in the next phase. The research approach and ultimate partnership with students to develop a tailored program are similar to other work that has been conducted in community settings. Pedagogical strategies and curricula have utilized the approach of conducting needs assessment prior to the collaborative development of curricula [37,38], as well as through partnerships with the target community to ultimately develop health-related programming [39,40,41,42].

Findings from the needs assessment data collected by the student research partners in Phase 1 were consistent across the two universities. The cross-sectional survey data mirrored that of previous work in that college students experience poor diet quality [43,44,45], high amounts of stress [46], and struggle with food security [47,48,49]. While health/wellness professionals across the two campuses referenced these health issues, they also were big proponents of health resources and programs that currently are available on campus that they feel students are underutilizing. This is consistent with previous work that highlights that a disconnect may exist between the perceived healthfulness of the campus environment and objective environmental healthfulness [50,51].

The process and results from this current research can serve as a guide to future educators and researchers who plan to address the health behaviors of college students. Using a CBPR approach to program planning allowed college students to identify the topics of need for a future proposed program. From their own examination of health and resources on campus, the student researchers were able to take ownership of a proposed plan to enhance the feasibility and buy-in of the project as it will be ultimately implemented for their community of peers on campus. This work can add to the CBPR literature by illustrating the feasibility and efficacy of weaving CBPR into an experiential learning undergraduate course and evidence-based program development. The students in the course “bought-in” to program planning and remained engaged in the development of the program even after the course ended.

Ultimately, based on the findings, the student research partners involved in this project, also the CAB, realized that a two-pronged program would be necessary to meet the health needs of their campuses. Both components of the program (hands-on cooking and text message campaign) are evidence-based intervention methods. Cooking and nutrition classes with college students have been shown to improve cooking confidence [51], and text messaging campaigns have proven to be effective methods for nudging people toward healthful behaviors [52]. The next steps in the present study will include pilot testing of the developed program. This will include a process and an outcome evaluation which will be designed by the CAB. The pilot program will consist of a “meal-prep” cooking class to address the health needs of improved diet quality and food security, along with, a text messaging campaign to share health-related resources and health programming activities occurring on the college campuses. Partnerships will be formed with student health-related organizations on campus to collate information to send out through this text message campaign. A subsequent article will provide more detail regarding the design of the final program and its outcomes.

Limitations. The current study has its limitations. The cohort of student research partners who enrolled in the 3-credit hour course, attended the workshop, and participated in the CAB was limited to 10 participants total. Though advertised as a course open to all fields of study, college students typically have a large series of major classes required for completion of their bachelor’s degrees; thus, fitting an additional 3-credit hour course into their already busy schedule can be difficult. Additionally, in Phase 1, the survey sample for the needs assessment was predominately white and female and so is not representative of college students on a national level. Future utilization of a course model may reduce credit hours offered to improve the feasibility of including the course in a busy schedule. Likewise, more work is needed to include a larger and more diverse sample in needs assessment data collection as well as the final determination of a campus-wide program.

## 5. Conclusions

By using a CBPR approach for health-related program planning, educators and researchers have a greater likelihood of addressing the current needs of the population they are targeting and developing a successful intervention to meet those health concerns. Equally important is training the next generation of community programmers to work with the specific communities they plan to intervene with to understand their needs and propose feasible and acceptable programming. Ultimately, this research aims to inspire college students to make positive changes to their environment and aid in making the college environment a more healthful place to learn, live, and work, in the hopes of improving the resiliency of the next generation of workers and the prevention of chronic disease.

## Figures and Tables

**Table 1:** Demographics Characteristics of the Phase 1 College Student Survey Sample (N = 1036).

Variable	n	Mean (SD), or %
Age, year	896	19.7 (1.6)
Body mass index	961	24.7 (5.1)
Gender		
Female	614	62.6
Male	316	32.3
Other	49	5.1
Race/Ethnicity		
White	885	90.4
Hispanic or Latino	29	3.0
Native American Black or African American	26	2.7
Other	28	2.8
Sexual Identity		
Heterosexual	692	70.8
Homosexual	43	4.4
Bisexual	143	14.6
Queer	37	3.8
Questioning/Unsure	35	3.6
Something else	28	2.9
Year in college		
Freshman	346	35.4
Sophomore	234	23.9
Junior	191	19.5
Senior	207	21.1
Dining plan		
Yes	507	51.8
No	471	48.2
Living situation		
On campus	500	51.2
Off campus	471	48.8

**Table 2:** Health Behaviors of the College Student Survey Sample.

Variable	Mean (SD), or % (n)
Perceived general health status (n = 971)	
Excellent	6.8 (66)
Very Good	19.9 (193)
Good	43.6 (423)
Fair	23.3 (226)
Poor	6.5 (63)
Perceived nutrition health status (n = 954)	
Excellent	5.1 (49)
Very good	15.2 (145)
Good	37.9 (362)
Fair	27.3 (260)
Poor	14.5 (138)
Hours of Sleep per night (n = 899)	6.9 (1.4)
Overall sleep quality (n = 976)	
Very good	9.2 (90)
Fairly good	62.4 (609)
Fairly bad	25.6 (250)
Very bad	2.8 (976)
Diet Quality (n = 983)	
Total Healthy Eating Index	47.6 (10.1)
Servings of Fruit	0.92 (0.35)
Servings of Vegetables	2.4 (0.52)
Cooking Confidence (n = 970)	
Cooking methods	3.8 (0.7)
Cooking with specific foods	4.0 (0.8)
Preparing recipes	4.0 (0.8)
Food Security (n = 981)	
High	38.6 (377)
Marginal	25.1 (245)
Low	19.9 (194)
Very Low	16.4 (160)
Perceived Stress (n = 970)	
Low	2.8 (27)
Moderate	54.9 (533)
High	42.3 (410)

**Table 3:** Nominal Group Technique Questions and Ranked Responses.

NGT Questions	Top 3 Choices in Ranking Order
If you were to participate in a health/well-being program on your campus, what would influence you to participate?	Incentives Convenience Personal Benefit
How often would you/others attend program events throughout a semester? Why?	1–2 times per month Once per week Twice per month
Probe: What time of day & day of the week do you believe would work best for events throughout the semester?	Weekday afternoon Weekday 3–4 p.m. Weekday after 4 p.m.
In ranking order, what top 3 topic areas/ideas for the intervention do you feel are most important to design for students at your university?	Communication of campus resources Nutrition knowledge and skills Mental health support
What top 2 modes of delivery do you feel would work best for our program? (in-person, online, text, mixture, etc.)	A mix of in-person and text Online More in-person
Overall, which idea from you and your peers’ presentations do you feel would be best received (people would be most excited to participate in) on your campus? Why?	Education (nutrition, budget, cooking) Sharing campus resources via text Incentive-based punch card

## Data Availability

For access to the data, please contact the corresponding author.
